# Differential Responses to Salt Stress in Four White Clover Genotypes Associated With Root Growth, Endogenous Polyamines Metabolism, and Sodium/Potassium Accumulation and Transport

**DOI:** 10.3389/fpls.2022.896436

**Published:** 2022-06-02

**Authors:** Zhou Li, Wan Geng, Meng Tan, Yao Ling, Yan Zhang, Liquan Zhang, Yan Peng

**Affiliations:** ^1^College of Grassland Science and Technology, Sichuan Agricultural University, Chengdu, China; ^2^Key Laboratory of Forage and Endemic Crop Biology, Ministry of Education, Inner Mongolia University, Hohhot, China

**Keywords:** ion transport, root to shoot ratio, plant growth regulator, salinization, differentially expressed genes, water use efficiency

## Abstract

Selection and utilization of salt-tolerant crops are essential strategies for mitigating salinity damage to crop productivity with increasing soil salinization worldwide. This study was conducted to identify salt-tolerant white clover (*Trifolium repens*) genotypes among 37 materials based on a comprehensive evaluation of five physiological parameters, namely, chlorophyll (Chl) content, photochemical efficiency of PS II (Fv/Fm), performance index on an absorption basis (PIABS), and leaf relative water content (RWC), and to further analyze the potential mechanism of salt tolerance associated with changes in growth, photosynthetic performance, endogenous polyamine metabolism, and Na^+^/K^+^ uptake and transport. The results showed that significant variations in salt tolerance were identified among 37 genotypes, as PI237292 and Tr005 were the top two genotypes with the highest salt tolerance, and PI251432 and Korla were the most salt-sensitive genotypes compared to other materials. The salt-tolerant PI237292 and Tr005 not only maintained significantly lower EL but also showed significantly better photosynthetic performance, higher leaf RWC, underground dry weight, and the root to shoot ratio than the salt-sensitive PI251432 and Korla under salt stress. Increases in endogenous PAs, putrescine (Put), and spermidine (Spd) contents could be key adaptive responses to salt stress in the PI237292 and the Tr005 through upregulating genes encoding Put and Spd biosynthesis (*NCA*, *ADC*, *SAMDC*, and *SPDS2*). For Na^+^ and K^+^ accumulation and transport, higher salt tolerance of the PI237292 could be associated with the maintenance of Na^+^ and Ca^+^ homeostasis associated with upregulations of *NCLX* and *BTB/POZ*. The K^+^ homeostasis-related genes (*KEA2*, *HAK25*, *SKOR*, *POT2*/*8*/*11*, *TPK3*/*5*, and *AKT1*/*5*) *are* differentially expressed among four genotypes under salt stress. However, the K^+^ level and K^+^/Na^+^ ratio were not completely consistent with the salt tolerance of the four genotypes. The regulatory function of these differentially expressed genes (DEGs) on salt tolerance in the white clover and other leguminous plants needs to be investigated further. The current findings also provide basic genotypes for molecular-based breeding for salt tolerance in white clover species.

## Introduction

Soil salinity has been decreasing global agricultural productivity, which poses a threat to agriculture and food security since more than 20% of agricultural land worldwide is negatively affected by soil salinization (Mickelbart et al., 2015). Identification or development of salt-tolerant germplasms or cultivars is one of the most effective strategies for mitigating salinity damage to crop productivity because genetic variation is often found within one particular species, especially for those widely distributed across the world (Munns, 2005). It has been reported that abundant genetic diversity and variation in salt tolerance among 552 sunflower (*Helianthus annuus*) germplasms and 30 materials with different genetic backgrounds were identified as salt-tolerant genotypes (Li et al., 2020a). A genome-wide association scan in 174 barley (*Hordeum vulgare*) accessions found that salt-tolerant germplasms exhibited stronger antioxidant capacity in response to salt stress (Thabet et al., 2021). A significant variation in salt tolerance was also observed among rice (*Oryza sativa*) germplasms (Yuan et al., 2020; Lar et al., 2021; Rasel et al., 2021). The salt tolerance of 15 basil (*Ocimum* spp.) genotypes varied with morphological parameters, growth, and photosynthetic performance (Shirahmadi et al., 2022). The white clover (*Trifolium repens*) is a vital legume component of the temperate pasture, which is cultivated widely as forage due to its high nutrient value. Improvement or maintenance of white clover productivity is essential for livestock husbandry under environmental stress (Egan et al., 2018). However, the white clover is often recognized as a salt-sensitive species. Salt stress is considered the main limitation to its production and quality in arid and semi-arid regions (Rogers et al., 1997; Wang et al., 2010). It is important to identify white clover genotypes with superior salt tolerance and productivity under salt stress.

Salt stress causes damage to plants mainly involved in osmotic stress, ion toxicity, and secondary stress, since high concentrations of salt ions in soil hinder water uptake, and the overaccumulation of Na^+^ in cells causes ion toxicity, physiological disturbance, nutritional imbalance, and oxidative damage (Motos et al., 2017). Intracellular ion homeostasis is a fundamental mechanism by which plants survive salt stress. However, the K^+^ uptake and transport are significantly inhibited by Na^+^ in plants, resulting in K^+^ deficiency under high salt conditions (Abbasi et al., 2016). K^+^ content has been considered to be a key indicator of salt tolerance because of its vital role in stress signaling, ion homeostasis, and nutrition (Wu et al., 2018). A previous study demonstrated that the maintenance of a higher K^+^/Na^+^ ratio was positively correlated with salt tolerance in different plant species. For example, salt-tolerant rice genotypes (Ghunsi, Nonabokra, and BINA dhan-10) exhibited a significantly higher K^+^/Na^+^ ratio than salt-sensitive BINA dhan-17, indicating that the K^+^/Na^+^ ratio could be used as an essential indicator of salt tolerance (Rasel et al., 2021). It has been found that the decrease in the in Na^+^/K^+^ ratio regulated by exogenous alpha-lipoic acid or transcription factor *OsSTAP1* through a transgenic approach was related to enhanced salt tolerance in plants (Wang Y. et al., 2020; Youssef et al., 2021). A study by Yan et al. (2020) found that melatonin application increased the K^+^/Na^+^ ratio and endogenous PAs accumulation, contributing to enhanced salt tolerance in rice. However, no significant differences in Na^+^ content, K^+^ content, and the Na^+^/K^+^ ratio were identified between salt-tolerant rice cultivar Zhegeng 78 and salt-sensitive cultivar Zhegeng 99 at post-germination and seedling stages under salt stress (Ye et al., 2021). In response to salt stress, salt-tolerant rice cultivar Pokkali exhibited significantly higher Na^+^ content and Na^+^/K^+^ ratio than salt-sensitive IR29, along with a significant upregulation of *OsNHX1*, which was responsible for the compartmentation of Na^+^ into vacuoles to maintain growth and photosynthetic performance under salt stress (Theerawitaya et al., 2020). These findings indicated that salt tolerance mechanism is related to the K^+^/Na^+^ ratio and that their contents could vary in different plant species.

In addition to Na^+^/K^+^ accumulation and transportation, salt tolerance is also involved in many other mechanisms such as osmotic adjustment, oxidation-reduction equilibrium, hormonal regulation, and metabolic homeostasis (Yu et al., 2020). It has been found that polyamine (PA) metabolic pathways were closely related to salt tolerance in plants (Pottosin et al., 2021). Putrescine (Put), spermidine (Spd), and spermine (Spm) are abundant PAs in plants exhibiting various roles in mediating stress tolerance as compatible solutes for osmotic adjustment, scavengers of reactive oxygen species for antioxidant defense, signal molecules for stress signal transduction, and regulators of ion channels for ion homeostasis (Minocha et al., 2014). Improvement in salt tolerance *via* enhanced PA biosynthesis and metabolism has been reported in many plant species such as wheat (Talaat, 2021) and switchgrass (*Panicum virgatum*) (Guan et al., 2020), and rice (Islam et al., 2020; Das et al., 2021). For the PA metabolism, arginine decarboxylase (ADC) catalyzes the decarboxylation of arginine into agmatine, which is used for the Put biosynthesis. Spd is produced from the Put by spermidine synthase (SPDS), and spermine synthase (SPMS) catalyzes Spm synthesis by using Spd. Two processes (from Put to Spd and from Spd to Spm) need the sequential addition of decarboxylated *S*-adenosyl-methionine synthesized by *S*-adenosylmethionine decarboxylase proenzyme (SAMDC). Polyamine oxidase (PAO) catalyzes the degradation of Spd and Spm in cells (Miller-Fleming et al., 2015). A previous study showed that overexpression of *SAMDC* or *ADC* could significantly improve salt tolerance in plants (Espasandin et al., 2018; Islam et al., 2020).

Enhanced salt tolerance in transgenic tobacco (*Nicotiana tabacum*) or apple (*Malus pumila*) plants *via* ectopic expression of a *CsTGase* or *MdATG8i* was associated with PA accumulation and Na^+^/K^+^ homeostasis (Huo et al., 2020; Zhong et al., 2020). However, different types of PAs may exhibit different responses to salt stress. For example, salt stress significantly increased Put content in salt-sensitive sorghum (*Sorghum bicolor*) plants, but a significant decline in Put content and significant increases in Spd and Spm contents in salt-tolerant plants (de Oliveira et al., 2020). It is worth further investigating the potential mechanism of salt tolerance associated with variations in endogenous PA metabolism and Na^+^/K^+^ transport in leguminous plants.

Salt tolerance is a complex trait and possesses species-specific regulatory effects and mechanisms in the plant kingdom (Van Zelm et al., 2020). Natural variations in PA metabolism and Na^+^/K^+^ accumulation and transport remain to be identified in white clover species. The aims of the current study were to screen and evaluate salt tolerance of 37 white clover germplasms and to elucidate the potential mechanism of salt tolerance among different genotypes associated with changes in growth, photosynthetic performance, endogenous PAs, and Na^+^/K^+^ accumulation. Transcriptional profiling further identified key genes involved in PAs metabolism and Na^+^/K^+^ transportation in four white clover genotypes with varying degrees of salt tolerance. The research results will help us gain an understanding of salt tolerance in leguminous plant species and provide basic materials for the salt tolerance breeding of the white clover.

## Materials and Methods

### Plant Material and Treatments

A total of 37 white clover genotypes were used for the evaluation of salt tolerance and subsequent research about the potential mechanism of genotypes differing in salt tolerance. Among them, 19 genotypes were provided by the National Plant Germplasm System of the United States. The 10 genotypes were commercial cultivars. The eight genotypes were wild resources collected from Sichuan in China (Supplementary Table 1). The seeds were germinated in petri dishes containing two layers of filter paper and 20 ml of deionized water for 8 days. The seedlings were then transplanted into polyvinyl chloride tubes (12 cm in diameter and 20 cm in length) filled with sand and loam (1: 1). Each tube contained five seedlings, and all tubes were placed in the greenhouse for 40 days for cultivation (average day/night temperatures of 24/17°C, 12 h photoperiod at 750 mmol m^–2^s^–1^ photosynthetically active radiation, and 65% relative humidity). A 250 ml of Hoagland’s nutrient solution was irrigated in each tube weekly (Hoagland and Arnon, 1950). For salt stress, all materials were divided into two groups: one group was cultivated under normal conditions without salt stress for 16 days, and the other group was irrigated with 200 ml of 150 mM NaCl for 3 days, 200 ml of 200 mM NaCl for 3 days, and 200 ml of 250 mM NaCl for 10 days (a total of 16 days of salt stress). The NaCl was dissolved in the Hoagland solution. Leaf and root samples were taken on the 16th day of normal cultivation and salt stress. Four independent replicates were used to determine the growth (fresh and dry weight) and physiological parameters, and three independent replicates were used to detect the transcriptome.

### Determinations of Growth Parameters and Water Status

The aboveground and underground fresh tissues of all plants in each tube were collected and weighed immediately to get a fresh weight. Then, these tissues were put into an oven at 105°C for 30 min and 75°C for 3 days until constant dry weight. Then, the dry to fresh weight ratio was calculated. The root to shoot ratio was calculated as the ratio of underground dry weight to aboveground dry weight. Relative water content (RWC) was evaluated according to the formula RWC = [(FW-DW)/(TW-DW)] × 100%. The FW, DW, or TW indicated fresh, dry, or turgid weight, respectively (Barrs and Weatherley, 1962).

### Determinations of Photosynthetic Parameters and Cell Membrane Stability

Cell membrane stability was evaluated by changing electrolyte leakage (EL) (Blum and Ebercon, 1981). Fresh leaves (0.15 g) were cleaned and immersed in 35 ml of deionized water for 24 h at 20°C in a 50 ml centrifuge tube. The initial conductivity (C1) of the solution was identified by using a conductivity meter (YSI Model 32, Yellow Springs Instrument Co., Yellow Spring, OH, United States). The centrifuge tube was autoclaved at 105°C for 15 min. After being cooled down to room temperature, the final conductivity (C2) was determined. Then, EL was calculated based on the formula EL (%) = C1/C2 × 100%. Chlorophyll (Chl) content was determined based on the method of Amnon (1949). For determination of photochemical efficiency of PS II (Fv/Fm) and performance index on an absorption basis (PIABS), a Chl fluorescence system (Pocket PEA, Hansatech, Norfolk, United Kingdom) was used. The PIABS, a key indicator of maximum photochemical efficiency and total numbers of activated photochemical reaction centers of PS II, reflects the health status of chloroplasts under stress conditions. A single layer of leaves was put into the leaf chamber for 20 min dark adaptation before the Fv/Fm and PI_ABS_ were recorded. Net photosynthetic rate (Pn), transpiration rate (Tr), stomatal conductance (Gs), intercellular CO_2_ concentration (Ci), and water use efficiency (WUE) were identified by using a portable photosynthesis system (CIRAS-3, PP Systems, Amesbury, MA, United States) that provided stable 400 μl L^–1^ CO_2_ and 800 μmol photon m^–2^ red and blue light.

### Determinations of Polyamines, Sodium/Potassium Contents, and Transcriptome

Endogenous PAs, Put, Spd, and Spm were identified by high-performance liquid chromatography (HPLC, Agilent-1200, Agilent Technologies, Santa Clara, CA, United States) with some modifications based on the method of Duan et al. (2008), which was demonstrated in our previous study (Li et al., 2016). To determine Na^+^ and K^+^ contents, an inductively coupled plasma-mass spectrometry (ICP-MS, ICAP6300, Thermo Fisher Scientific, MA, United States) was used, and the assay method in detail has been recorded in the study by Geng et al. (2021). The transcriptome was used to detect differentially expressed genes (DEGs) in the leaves of the white clover in response to salt stress. RNA extraction, library construction, and sequencing have also been demonstrated in our previous study (Li et al., 2017). The gene function was annotated by using databases including Nr (NCBI non-redundant protein sequences), NCBI non-redundant nucleotide sequences (Nt), clusters of orthologous groups of proteins (KOG/COG), a manually annotated and reviewed protein sequence database (Swiss-Prot), KEGG Ortholog database (KO), and gene ontology (GO). The *p*-value < 0.05 and fold change of gene ≥ 1.5 were set as the threshold for DEGs.

### Statistical Analysis

For statistical analysis of growth and physiological parameters, differences among treatment means were tested using Fisher’s protected least significance (LSD) test at the *P* ≤ 0.05 probability level. For a comprehensive evaluation of salt tolerance, subordinate function value analysis (SFVA) of five physiological parameters (EL, Chl, Fv/Fm, PIABS, and RWC) was performed. Then, the *D* value was calculated using the SFV (*D* = ΣU (X_i_)/n). The bigger the *D* value, the higher salt tolerance (Li Z. et al., 2021). A heat map of physiological parameters was created using the R statistical software (R3.5.2 by the R Development Core Team).

## Results

### Evaluation of Salt Tolerance Among 37 White Clover Genotypes

The heat map showed physiological parameters: EL, Chl, Fv/Fm, PIABS, and RWC; they were changed by salt stress in 37 materials (Supplementary Figure 1A). These materials could be mainly divided into three subclusters based on the hierarchical clustering, as subcluster a included 19 materials, subcluster b included 11 materials, and subcluster c contained seven materials (Supplementary Figure 1A). The salt tolerance of 37 materials was ranked according to the SFVA and *D* values (Supplementary Figure 1B). As shown in Supplementary Figure 1B, the top five materials with higher salt tolerance than other materials in the sequences were PI237292, Tr005, Sulky, Tr002, and Tr037. Korla (least) and PI251432 (the last but one) exhibited less salt tolerance than other materials (Supplementary Figure 1B).

### Water Status and Photosynthetic Parameters Affected by Salt Stress Among Four White Clover Genotypes

**FIGURE 1:**
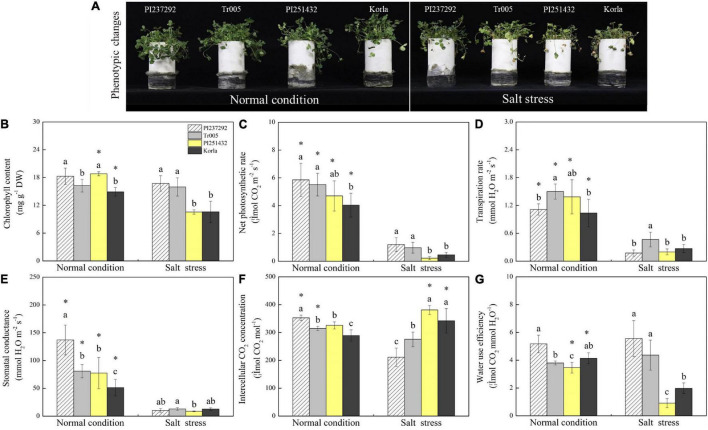
Changes in **(A)** phenotypic changes, **(B)** chlorophyll content, **(C)** net photosynthetic rate, **(D)** transpiration rate, **(E)** stomatal conductance, **(F)** intercellular CO2 concentration, and **(G)** water use efficiency in the leaves of four white clover materials (PI237292, Tr005, PI251432, and Korla) under normal and salt stress conditions. Vertical bars indicate ± standard error (SE) of the mean (*n* = 4), and different letters above the column indicate significant differences (*P* ≤ 0.05) under normal conditions or salt stress. The “*” represents the significant difference between normal conditions and salt stress for a specific genotype (PI237292, Tr005, PI251432, or Korla).

Phenotypic changes showed that salt stress caused leaf wilting and chlorosis, and PI251432 and Korla suffered from severer damage than PI237292 and Tr005 in response to salt stress (Figure 1A). EL increased significantly, whereas RWC decreased greatly in the leaves of PI237292, Tr005, PI251432, and Korla under salt stress (Supplementary Figures 2A,B). PI237292 and Tr005 maintained significantly lower EL and higher RWC than PI251432 and Korla after 16 days of salt stress (Supplementary Figures 2A,B). No significant differences in Fv/Fm and PIABS were observed among the four materials under normal conditions (Supplementary Figures 2C,D). Salt stress caused a significant decline in Fv/Fm in the leaves of PI237292, PI251432, or Korla, but not in Tr005 (Supplementary Figure 2C). Under salt stress, PI237292 and Tr005 exhibited more than 3 or 16 times higher PIABS than PI251432 or Korla, respectively (Supplementary Figure 2D). Chl content was maintained at an average level in PI237292 and Tr005 when subjected to salt stress but significantly declined in PI251432 or Korla (Figure 1B). Pn, Tr, and Gs were reduced significantly in four materials under salt stress (Figures 1C–E). Ci significantly decreased in PI237292 and Tr005 but increased in PI251432 or Korla under salt stress (Figure 1F). A prolonged period of salt stress did not affect WUE significantly in the leaves of PI237292 and Tr005 but caused a significant decline in WUE in the leaves of PI251432 or Korla (Figure 1G).

### Growth Affected by Salt Stress Among Four White Clover Genotypes

**FIGURE 2:**
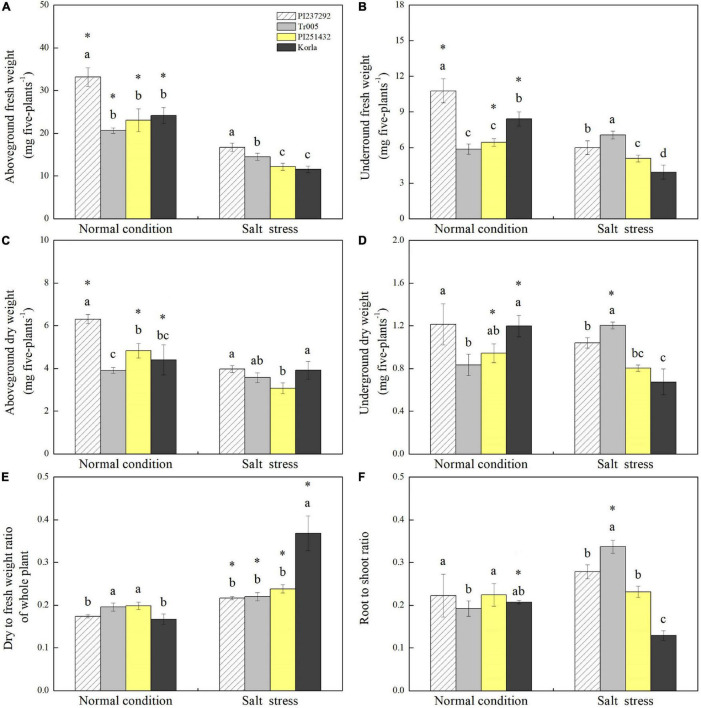
Changes in **(A)** aboveground fresh weight, **(B)** underground fresh weight, **(C)** aboveground dry weight, **(D)** underground dry weight, **(E)** dry to fresh weight ratio, and **(F)** root to shoot ratio in the leaves of four white clover materials (PI237292, Tr005, PI251432, and Korla) under normal and salt stress conditions. Vertical bars indicate ± standard error (SE) of the mean (*n* = 4), and different letters above the column indicate significant differences (*P* ≤ 0.05) under normal conditions or salt stress. The “*” represents the significant difference for a specific genotype (PI237292, Tr005, PI251432, or Korla) between normal conditions and salt stress.

PI237292 had the highest aboveground fresh weight of the three materials under normal conditions and salt stress (Figure 2A). Tr005 increased its aboveground fresh weight by 25% more than PI251432 or Korla under salt stress (Figure 2A). Salt stress led to a significant decline in the underground fresh weight of PI237292, PI251432, or Korla but did not affect the underground fresh weight of Tr005 (Figure 2B). PI237292 had more than a 30% increase in aboveground dry weight than the other three materials under normal conditions but did not maintain the superiority under salt stress (Figure 2C). Significant declines in aboveground dry weight were caused by salt stress in PI237292, PI251432, and Korla, but not in Tr005 (Figure 2C). There was no significant difference in the underground dry weight of PI237232 under normal conditions and salt stress (Figure 2D). Salt stress significantly improved underground dry weight in Tr005 but decreased underground dry weight in PI251432 and Korla (Figure 2D). Salt-caused increase in dry to fresh weight ratio was more pronounced in Korla than in other materials (Figure 2E). Under salt stress, the root to shoot ratio was highest in Tr005, second highest in PI237292, third highest in PI251432, and lowest in Korla (Figure 2F). In addition, a significant decline in the root to shoot ratio was only observed in Korla under salt stress (Figure 2F).

### Endogenous Polyamines, Sodium, and Potassium Content Were Affected by Salt Stress Among Four White Clover Genotypes

**FIGURE 3:**
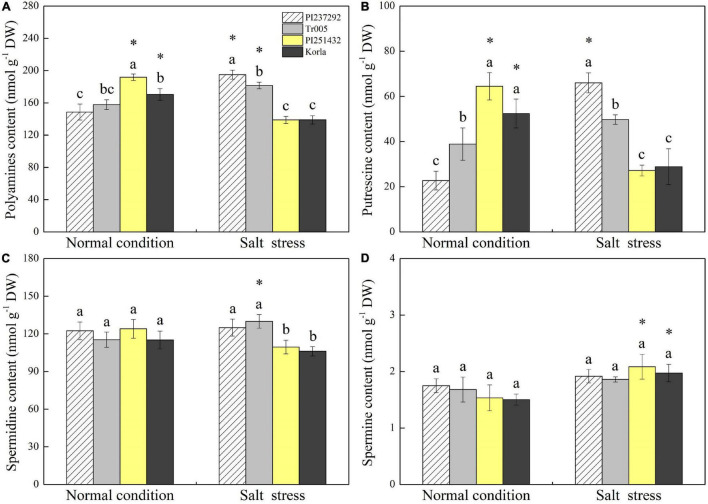
Changes in **(A)** polyamine content, **(B)** putrescine content, **(C)** spermidine content, and **(D)** spermine content in the leaves of four white clover materials (PI237292, Tr005, PI251432, and Korla) under normal and salt stress conditions. Vertical bars indicate ± standard error (SE) of the mean (*n* = 4), and different letters above the column indicate significant differences (*P* ≤ 0.05) under normal conditions or salt stress. The “*” represents the significant difference for a specific genotype (PI237292, Tr005, PI251432, or Korla) between normal conditions and salt stress.

**FIGURE 4:**
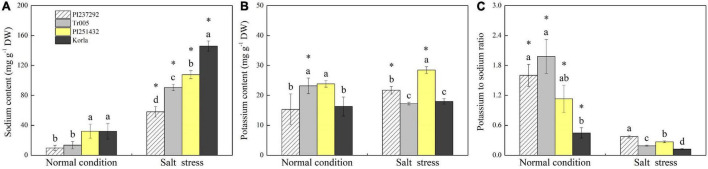
Changes in **(A)** sodium content, **(B)** potassium content, and **(C)** potassium to sodium ratio in the leaves of four white clover materials (PI237292, Tr005, PI251432, and Korla) under normal and salt stress conditions. Vertical bars indicate ± standard error (SE) of the mean (*n* = 4), and different letters above the column indicate significant differences (*P* ≤ 0.05) under normal conditions or salt stress. The “*” represents the significant difference for a specific genotype (PI237292, Tr005, PI251432, or Korla) between normal conditions and salt stress.

Endogenous PAs content was highest in PI251432 than in the other three materials under normal conditions (Figure 3A). Salt stress caused a significant increase in PAs content in PI237292 and Tr005 but a significant decrease in PI251432 and Korla (Figure 3A). PI251432 and Korla maintained significantly higher Put content than PI237292 and Tr005 under normal conditions; on the contrary, PI237292 and Tr005 showed significantly higher Put content than PI251432 and Korla did in response to salt stress (Figure 3B). No significant differences were observed in Spd and Spm contents among four materials under the normal condition as well as in Spm content between four materials under salt stress (Figures 3C,D). PI251432 and Korla exhibited a 17% increase in Spd content compared to PI251432 and Korla under salt stress (Figure 3C). A significant rise in Na^+^ content was caused by salt stress in the leaves of four materials, as demonstrated by the highest content in Korla, the second highest content in PI251432, the third highest in Tr005, and the lowest in PI237292 (Figure 4A). Under normal conditions, Tr005 and PI251432 exhibited significantly higher K^+^ content than PI237292 and Korla, and PI251432 had the highest K^+^ content than other materials under salt stress (Figure 4B). Salt stress resulted in a significant K^+^ to Na^+^ ratio decline in four materials (Figure 4C). PI237292 or Korla maintained the highest or the lowest K^+^ to Na^+^ ratio than other materials under salt stress, respectively (Figure 4C).

### Differentially Expressed Genes Affected by Salt Stress Among Four White Clover Genotypes

**FIGURE 5:**
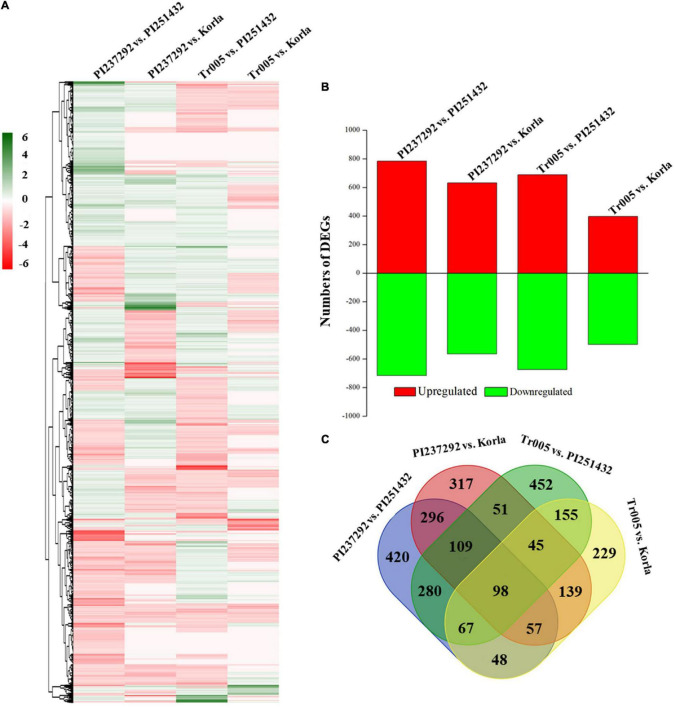
Changes in the **(A)** heat map of differentially expressed genes (DEGs), **(B)** the total number of DEGs, and **(C)** the Venn diagram of DEGs of each comparable group based on the transcriptome analysis.

Supplementary Table 2 shows basic information about the transcriptome data, namely, samples, replicates, clean reads, clean bases, GC content, and % ≥ Q30. Transcriptome identified abundant genes in the four materials (PI237292, Tr005, PI251432, and Korla) in response to salt stress, and analyses of the GO, KEGG, and KOG of these identified genes involved in gene function and metabolic processes are depicted in Supplementary Figures 3–5. The heat map of DEGs demonstrated different comparable groups, including PI237292 vs. PI251432, PI237292 vs. Korla, Tr005 vs. PI251432, and Tr005 vs. Korla, and had differential DEGs profiling (Figure 5A). The most abundant or least DEGs were identified in PI237292 vs. PI251432 or Tr005 vs. Korla, respectively (Figure 5B). The Venn diagram of DEGs showed total numbers of common and differential DEGs in four comparable groups (Figure 5C).

**FIGURE 6:**
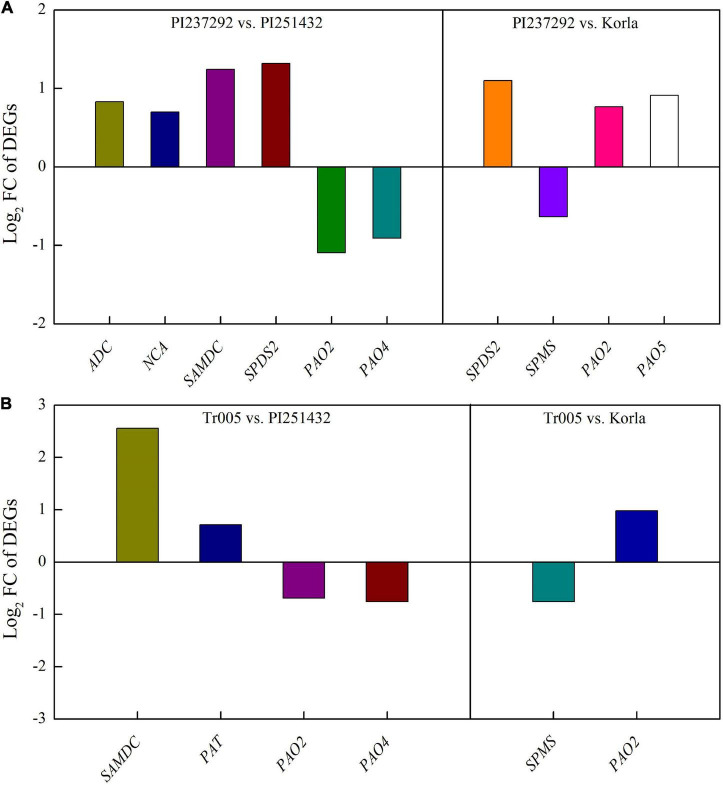
Changes in key differentially expressed genes in **(A)** PI237292 vs. PI251432 and PI237292 vs. Korla and **(B)** Tr005 vs. PI251432 and Tr005 vs. Korla involved in polyamine metabolism in the leaves of white clover under salt stress. *ADC*, arginine decarboxylase; *NCA*, *N*-carbamoylputrescine amidase; *SAMDC*, *S*-adenosylmethionine decarboxylase proenzyme; *SPDS2*, spermidine synthase 2; *PAO2*, polyamine oxidase 2; *PAO4*, polyamine oxid*a*se 4; *PAO5*, polyamine oxidase 5; SPMS, spermine synthase; *PAT*, polyamine transporter.

**FIGURE 7:**
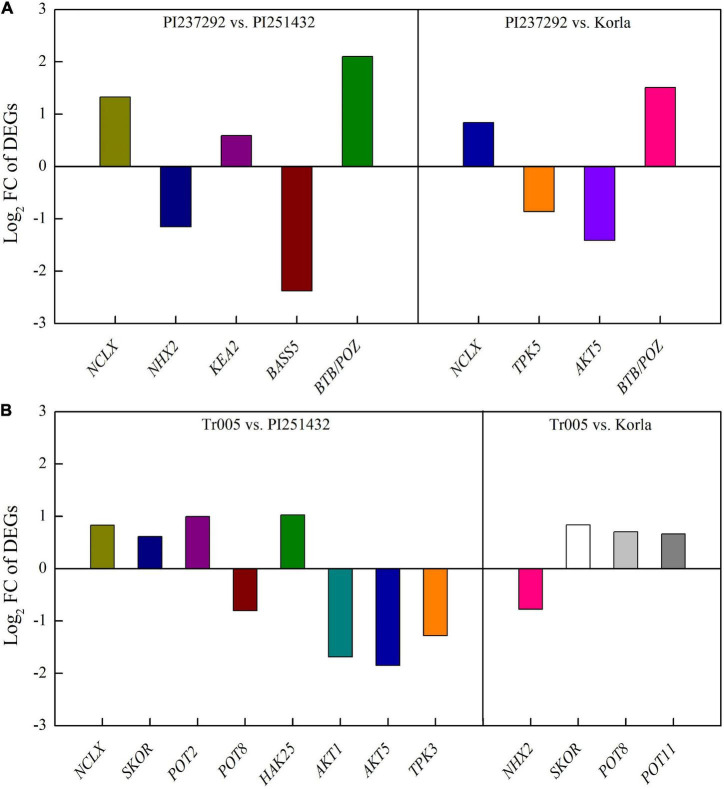
Changes in key differentially expressed genes in **(A)** PI237292 vs. PI251432 and PI237292 vs. Korla and **(B)** Tr005 vs. PI251432 and Tr005 vs. Korla involved in sodium and potassium transport in the leaves of white clover under salt stress. *NCLX*, sodium/calcium exchanger; *NHX2*, sodium/hydrogen exchanger 2; *KEA2*, potassium efflux antiporter 2; *BASS5*, probable sodium/metabolite cotransporter; *BTB/POZ*, BTB/POZ domain-containing protein; *TPK3*, two-pore potassium channel 3; *TPK5*, two-pore potassium channel 5; *AKT1*, potassium channel 1; *AKT5*, potassium channel 5; *SKOR*, potassium channel SKOR; *POT2*, potassium transporter 2; *POT8*, potassium transporter 8; *POT11*, potassium transporter 11; *HAK25*, high affinity potassium transporter 25.

For DEGs involved in PAs metabolism, four DEGs (*ADC*, *NCA*, *SAMDC*, and *SPDS2*) were significantly upregulated, and two DEGs (*PAO2* and *PAO4*) were significantly downregulated in the PI237292 vs. PI251432 (Figure 6A). *SPDS2*, *PAO2*, and *PAO5* were upregulated, but *SPMS* was downregulated in the PI237292 vs. Korla (Figure 6A). In the Tr005 vs. PI251432, two DEGs (*SAMDC* and *PAT*) were upregulated, and two (*PAO2* and *PAO4*) were downregulated, and *SPMS* or *PAO2* was downregulated or upregulated in the Tr005 vs. Korla, respectively (Figure 6B). For DEGs involved in Na^+^ and K^+^ transportation, three DEGs (*NCLX*, *KEA2*, and *BTB/POZ*) or two DEGs (*NHX2* and *BASS5*) were upregulated or downregulated in PI237292 vs. PI251432, respectively (Figure 7A). PI237292 exhibited significantly higher *NCLX* and *BTB/POZ* as well as lower *TPK5* and *AKT5* under salt stress (Figure 7A). Significant upregulation in four DEGs (*NCLX*, *SKOR*, *POT2*, and *HAK25*) was identified in the Tr005 vs. PI251432, and another four DEGs (*POT8*, *AKT1*, *AKT5*, and *TPK3*) were downregulated (Figure 7B). Under salt stress, Tr005 significantly upregulated *SKOR*, *POT8*, and *POT11* while downregulating *NHX2* compared to Korla (Figure 7B).

## Discussion

Increasing soil salinization yearly at a rate of 10% accelerates the demand for salt-tolerant crop germplasms to maintain agricultural yield worldwide (Shrivastava and Kumar, 2015). For the evaluation of salt tolerance in white clover species, the study by Rogers et al. (1994) found that cv. Haifa and cv. Irrigation exhibited superior salt tolerance to the other four cultivars, Aran, Kopu, Pitau, and Tamar. Wang et al. (2010) identified quantitative trait loci for salinity tolerance in the white clover population comprising 232 progenies. However, salt tolerance often varies depending on plant species and cultivars. The primary symptoms of salt damage include tip burning, leaf yellowing and rolling, plant wilting, and stunted plant growth (Acosta-Motos et al., 2017). EL, Chl, or Fv/Fm have been widely used to identify tolerance to environmental stress in various plant species, such as heat stress in creeping bentgrass (Li Z. et al., 2021), drought tolerance in common bean (*Phaseolus vulgaris*) genotypes (Munyasa, 2013), and salt tolerance in blue panicgrass (*Panicum antidotale*) (Javed et al., 2021), basil (Shirahmadi et al., 2022), and tomato (*Lycopersicon esculentum*) (Sivakumar et al., 2020). Recently, PIABS has been regarded as a potential indicator of stress tolerance since it synthetically reflects the maximum photochemical efficiency of PSII and is more sensitive than the Fv/Fm in response to diverse abiotic stresses (Dai et al., 2019; Liang et al., 2021; Shirahmadi et al., 2022). Our current study demonstrated physiological variations in 37 white clover materials under salt stress. PI237292 and Tr005 exhibited superior salt tolerance with high levels of RWC, Fv/Fm, and PIABS, as well as lower EL levels than other white clover materials. On the contrary, PI251432 and Korla were the most susceptible to salt stress. These salt-tolerant and salt-sensitive white clovers provide potential materials for breeding, cultivation, and utilization and further studies on the mechanism of salt tolerance in leguminous plants.

One of the harmful effects caused by salt stress is photosynthetic inhibition due to limited water supply, weakened gaseous interchange, and accelerated chloroplast degradation (Acosta-Motos et al., 2017). In general, the limitation of photosynthesis could be caused by the stomata factor, mainly owing to reduced stomatal conductance and limited intercellular CO_2_ supply and non-stomatal factor as a result of chloroplast degradation and impaired photobiochemistry under stressful conditions (Pan et al., 2021). A previous study demonstrated that photosynthesis in sunflower (*H. annuus*) was not affected by non-stomatal limitation under mild salt stress (Steduto et al., 2000). The salt-caused decline in Pn was ascribed to the stomatal limitation in sorghum (Yan et al., 2012). Significant decreases in Pn, Gs, and Ci were observed in PI237292 and Tr005 in response to salt stress, but Chl contents were not significantly affected by salt stress in these two white clover materials, which implied that stomatal limitation dominated the main limiting factor to photosynthesis in the current study. However, salt stress caused significant decreases in Chl content, Pn, and Gs while significantly increasing Ci in PI251432 and Korla, indicating that PI251432 and Korla have already not effectively used intercellular CO_2_ for chloroplast degradation and impaired photobiochemistry, as mainly affected by non-stomatal limitation. In addition, WUE is an essential indicator of energy conversion efficiency, reflecting homeostasis between Pn and Tr (Lavergne et al., 2019). Maintenance of better WUE could be attributed to better water utilization and growth associated with enhanced salt tolerance in plants (Lugassi et al., 2019; Oljira et al., 2020). Therefore, PI237292 and Tr005 exhibited significantly higher WUE than PI251432 and Korla under salt stress, which could be a critical reason for survival.

Salt stress inhibits plant photosynthetic rate, resulting in reduced biomass accumulation. Maintenance of yield stability is a major challenge for many mesophytes, including white clover, under salt stress conditions (Rogers et al., 1994; Guo et al., 2013; Javed et al., 2021). The previous study showed that salt stress significantly caused declines in chlorophyll content and biomass of white clover (Han et al., 2014). Salt-tolerant rice genotypes grew better than salt-sensitive plants under salt stress (Rasel et al., 2021). When plants suffer from salt stress, alteration in root to shoot ratio indicates allocation strategy of biomass. Increased root to shoot ratio is attributed to salt tolerance since roots are in close contact with salt ions and are responsible for water uptake and nutrient absorption (Maggio et al., 2007; da Silva et al., 2008). An earlier study showed that tomato cultivars Arka Samrat and Arka Pakshak, with stronger salt tolerance, had longer root length and higher root dry weight than salt-sensitive PKM-OP (Sivakumar et al., 2020). Rice genotypes exhibited higher salt tolerance, as evidenced by the alleviation of growth limitation of roots and shoots under salt stress (Rasel et al., 2021). Denser root systems caused by arbuscular mycorrhizal in two maize genotypes (salt-tolerant JD52 with a large root system and salt-sensitive FSY1 with a small root system) were propitious for the amelioration of salinity damage (Wang et al., 2021). This present study found that PI237292 and Tr005 grown in salt conditions maintained significantly higher underground dry weight and root to shoot ratio than PI251432 and Korla, indicating that the mediation of root growth and root to shoot ratio could confer better tolerance to salt stress. An increase in the root to shoot ratio regulated by paclobutrazol was also reported in barley cultivars associated with enhanced salt tolerance (Özmen et al., 2003). These findings further proved that enhanced root growth and allocation could be beneficial for the improvement of salt tolerance associated with moisture and nutrition absorption.

Plants develop various adaptive strategies against salt stress, such as alterations in defense-associated compounds and PAs (Gupta et al., 2013). It has been well documented that regulation of PA accumulation and metabolism improved tolerance against various types of environmental stresses, including salt stress, by triggering many biochemical reactions in white clover and other plant species (Li et al., 2020b,d; Geng et al., 2021). In response to salt stress, total PAs and Put significantly accumulated in salt-tolerant PI237232 and Tr005 but significantly declined in salt-sensitive PI251432 and Korla compared to normal conditions. PI237232 and Tr005 also exhibited significantly higher endogenous Spd content than PI251432 and Korla, but no significant difference in Spm content was observed among the four materials under salt stress. These findings were consistent with the results from the transcriptome, as PI237232 and Tr005 significantly upregulated genes encoding Put and Spd biosynthesis (*NCA*, *ADC*, *SAMDC*, and *SPDS2*) and PAs transport (*PAT*) but downregulated Spm biosynthesis-related gene *SPMS* relative to PI251432 and Korla. It has been reported that transgenic plants overexpressing *SAMDC*, *ADC*, and *SPDS* improved salt tolerance in different plant species by increasing endogenous PA content (He et al., 2008; Espasandin et al., 2018; Islam et al., 2020; Jia et al., 2021). NCA is an enzyme involved in the degradation of N-carbamoylputrescine into Put (Takahashi and Tong, 2015). Increased Put biosynthesis through inducing NCA accumulation could mitigate detrimental effects of oxidative damage (Wang et al., 2022). PAT is responsible for PA transport (Fujita et al., 2012; Mulangi et al., 2012). Although PAT was involved in modulating heat tolerance in *Arabidopsis* by enhancing mRNA stability (Shen et al., 2016), its role in regulating salt tolerance remains unclear and requires further investigation in plants. However, expression levels of PAs degradation-related genes (*PAO2*, *PAO4*, and *PAO5*) differed between PI237292/Tr005 vs. PI251432 and PI237292/Tr005 vs. Korla in response to salt stress. The current findings highlight the significant roles of endogenous Put and Spd as key adaptive regulators in white clover suffering from salt stress.

The PAs were also involved in the regulation of Na^+^ homeostasis, contributing to enhanced salt tolerance in plants. For example, PAs mitigated salt-caused damage owing to their biological functions in improving the K^+^/Na^+^ ratio and Na^+^ compartmentalization in creeping bentgrass (Geng et al., 2021). A previous study also found that causative variation in the Na^+^/K^+^ ratio had a dominant position on salt tolerance in 369 tomato accessions (Wang Z. et al., 2020). Significant variations in Na^+^/K^+^ content and ratio were observed in six wheat (*Triticum aestivum*) genotypes (Iqra et al., 2020). An increase in the K^+^/Na^+^ ratio through exogenous application of *Azospirillum brasilense* or silicon contributing to enhanced salt tolerance has been reported in the white clover (Guo et al., 2013; Khalid et al., 2017). Our current findings demonstrated that salt-tolerant PI237292 maintained a significantly higher K^+^/Na^+^ ratio than salt-tolerant Tr005 and salt-sensitive PI251432 and Korla in response to salt stress; however, a significantly higher K^+^/Na^+^ ratio was identified in salt-sensitive PI251432 than in salt-tolerant Tr005 and salt-sensitive Korla, which indicated that salt tolerance in white clover genotypes not only depended on K^+^/Na^+^ ratio but also involved other mechanisms.

Transcriptional profiling identified many DEGs related to Na^+^ transport and compartmentalization. Among these DEGs, the BTB/POZ domain-containing protein family has diverse functions of stress-defensive protection, namely, transcriptional regulation, ion transportation, and protein degradation (Tazuke et al., 2015; Li et al., 2018; Zhang et al., 2021). Transgenic *Arabidopsis* overexpressing a novel BTB domain-containing protein gene *AtSIBP1* enhanced tolerance to salt stress (Wan et al., 2019). *NCLX* encoding Na^+^/Ca^2+^ exchanger protein plays an important role in Ca^2+^ homeostasis in plants under stress conditions (Singh et al., 2015). Higher salt tolerance in the PI237292 could be associated with the maintenance of Na^+^ and Ca^+^ homeostasis through the activation of *NCLX* and *BTB/POZ* under salt stress. The NHXs family is recognized as Na^+^/H^+^ exchangers to sequestrate cytoplasmic Na^+^ into the vacuole, which is a critical regulatory pathway for plants to decrease Na^+^ concentration in the cytosol (Long et al., 2020). Enhanced Na^+^ compartmentalization by upregulating *NHXs* was related to higher salt tolerance in plants (Geng et al., 2020; Geng et al., 2021). Our previous study found that γ-aminobutyric acid improved salt tolerance of creeping bentgrass by decreasing Na^+^ accumulation in the leaves along with significant inhibition of *AsNHX1*, *AsNHX2*, *AsNHX4*, and *AsNHX6* expression (Li et al., 2020c). Similar findings were presented in our present results. The PI237232 and the Tr005 accumulated significantly lower Na^+^ content and *NHX2* expression level than the PI251432 and Korla under salt stress. The “the more Na^+^ accumulation, the higher *NHX2* expression level in cells” could be an important adaptive response when plants suffer from salt stress since the transportation of Na^+^ from the cytosol to the vacuole is an effective approach to decreasing Na^+^ toxicity to plant cells. It has been proved that Na^+^ accumulation in vacuoles could act as a cheap strategy for osmotic adjustment in plants under high salinity stress (Glenn and Brown, 1998; Wang et al., 2004). *BASS* serves as a Na^+^/metabolite cotransporter, which could be significantly caused by salt stress (Huang et al., 2018; Şahin-Çevik et al., 2020). However, there is only limited information available about the function and regulatory mechanism of salt tolerance in plants.

Many DEGs involved in K^+^ homeostasis were identified in four white clover genotypes under salt stress, including *KEA2*, *HAK25*, *SKOR*, *POT2*/*8*/*11*, *TPK3*/*5*, and *AKT1*/*5*. The KEAs family regulates intracellular K^+^ homeostasis for ameliorating salt toxicity in plants. It has been reported that the inhibition of *GhKEA4* and *GhKEA12* expression led to salt sensitivity in cotton (*Gossypium*) plants (Li Y. et al., 2021). *Arabidopsis KEA2* overexpression conferred salt tolerance in yeast (*Saccharomyces cerevisiae*) cells (Aranda-Sicilia et al., 2012). HAKs serve as high-affinity K^+^ transporters involved in K^+^ uptake and K^+^/Na^+^ homeostasis in plants under salt stress (Chen et al., 2015; Shen et al., 2015). SKORs are involved in the uptake and transport of K^+^ from roots to leaves, conferring salt tolerance in plants (Long-Tang et al., 2018). *POTs* encode K^+^ transporter proteins in plants (Shabala and Cuin, 2008). TPKs are vacuolar two-pore K^+^ channel proteins and are responsible for the release of vacuolar K^+^ to the cytosol, which plays a critical role in K^+^ homeostasis (Gobert et al., 2007). An earlier study found that enhanced salt tolerance in transgenic tobacco with *PaTPK1* overexpression could be associated with the improvement in K^+^ transfer from the vacuole to the cytosol, thereby maintaining cytosolic K^+^ homeostasis (Wang et al., 2013). AKTs are known as inward-rectifying K^+^ channels, and their expression affects K^+^ uptake and nutrition (Hirsch et al., 1998; Ahmad et al., 2016). Salt stress significantly inhibited *OsAKT1* expression in the salt-tolerant rice variety Pokkali with less Na^+^ accumulation but not in the salt-sensitive variety IR29 with extremely high Na^+^ accumulation. However, there was no significant difference in K^+^ content between these two varieties under salt stress (Golldack et al., 2003). The study of Ahmad et al. (2016) also found that either loss of *OsAKT1* function rice mutant or transgenic rice overexpressing the *OsAKT1* did not show the difference in salt tolerance as compared to wild type, but *OsAKT1* expression increased Na^+^ accumulation and Na^+^/K^+^ ratio suggesting its role in Na^+^ uptake under salt stress. In the current study, the K^+^ homeostasis-related genes (*KEA2*, *HAK25*, *SKOR*, *POT2*/*8*/*11*, *TPK3*/*5*, and *AKT1*/*5*) were differentially expressed among four white clover genotypes. The K^+^ level and K^+^/Na^+^ ratio were not completely consistent with the salt tolerance of four white clover genotypes. Therefore, the regulatory function and mechanism of these DEGs associated with salt tolerance in the white clover and other leguminous plants need to be investigated further.

## Conclusion

Salt-tolerant and salt-sensitive white clover genotypes were identified among 37 genotypes based on the evaluation of physiological traits (EL, Chl, Fv/Fm, PIABS, and RWC), as PI237232 and Tr005 were the top two genotypes with the highest salt tolerance, and PI251432 and Korla were the most salt-sensitive genotypes compare to other materials. In response to salt stress, the salt-tolerant PI237232 and Tr005 not only maintained significantly lower EL but also showed significantly better photosynthetic performance, higher leaf RWC, underground dry weight, and root to shoot ratio than the salt-sensitive PI251432 and Korla. Increases in endogenous PAs, Put, and Spd contents could be key adaptive responses to salt stress in the PI237232 and the Tr005 through upregulating genes encoding Put and Spd biosynthesis (*NCA*, *ADC*, *SAMDC*, and *SPDS2*). For Na^+^ and K^+^ accumulation and transport, higher salt tolerance of the PI237292 could be associated with the maintenance of Na^+^ and Ca^+^ homeostasis through the activation of *NCLX* and *BTB/POZ*. The K^+^ homeostasis-related genes (*KEA2*, *HAK25*, *SKOR*, *POT2*/*8*/*11*, *TPK3*/*5*, and *AKT1*/*5*) are differentially expressed among four genotypes under salt stress. However, the salt tolerance of four genotypes was not completely consistent with the higher K^+^ level and K^+^/Na^+^ ratio. The regulatory function of these DEGs on salt tolerance in the white clover and other leguminous plants needs to be investigated further.

## Data Availability Statement

The datasets presented in this study can be found in online repositories. The names of the repository/repositories and accession number(s) can be found in the article/Supplementary Material.

## Author Contributions

ZL, YP, and LZ conceived and designed the experiments. ZL and YP provided different chemical reagents and experimental materials. WG, MT, and ZL performed the experiment, analyzed the data, and wrote the manuscript. YL, YZ, LZ, and YP reviewed and improved the manuscript. All authors have read and approved the published version of the manuscript.

## Conflict of Interest

The authors declare that the research was conducted in the absence of any commercial or financial relationships that could be construed as a potential conflict of interest.

## Publisher’s Note

All claims expressed in this article are solely those of the authors and do not necessarily represent those of their affiliated organizations, or those of the publisher, the editors and the reviewers. Any product that may be evaluated in this article, or claim that may be made by its manufacturer, is not guaranteed or endorsed by the publisher.
