# Supplementary material for: Differential Responses to Salt Stress in Four White Clover Genotypes Associated With Root Growth, Endogenous Polyamines Metabolism, and Sodium/Potassium Accumulation and Transport
Source: Front Plant Sci. 2022 Jun 2;13:896436. doi: 10.3389/fpls.2022.896436 (PMC9201400; doi:10.3389/fpls.2022.896436)
Supplement: Supplementary file 1 [file Data_Sheet_1.docx]

**Table S1** Cultivar name or accession number of NPGS and their origins of all white clover materials used in this study.

| Cultivar name or Accession number | Origin | Cultivar name or Accession number | Origin |
| --- | --- | --- | --- |
| PI227876 | Iran | Tr 002 | China |
| PI234678 | France | Tr 005 | China |
| PI234840 | Germany | Tr 033 | China |
| PI237292 | Denmark | Tr 037 | China |
| PI239978 | Spain | Tr 039 | China |
| PI249873 | Greece | Tr 059 | China |
| PI251432 | Croatia | Tr 077 | China |
| PI251862 | Austria | Tr 090 | China |
| PI288084 | Ireland | Beber | Denmark |
| PI418912 | Italy | Zapican | Argentina |
| PI419302 | Greece | Haifa | Australia |
| PI419304 | Greece | Harmony | / |
| PI419316 | Greece | Pixie | America |
| PI419459 | Switzerland | Korla | Australia |
| PI419580 | Japan | Ladino | America |
| PI494747 | Romania | MAG | Argentina |
| PI512040 | America | Miracle | / |
| PI542904 | Croatia | Sulky | Argentina |
| PI634148 | Kazakhstan |  |  |

**Table S2** Cultivar name or accession number of NPGS and their origins of all white clover materials used in this study.

| Conditions | Samples | Clean reads | Clean bases | GC content | %≥Q30 |
| --- | --- | --- | --- | --- | --- |
| Normal condition | PI237292-1 | 21,717,348 | 6,502,944,742 | 42.10% | 93.84% |
|  | PI237292-2 | 21,187,938 | 6,338,930,896 | 41.87% | 93.18% |
|  | PI237292-3 | 19,533,136 | 5,833,540,166 | 43.14% | 94.22% |
|  | Tr005-1 | 19,345,970 | 5,766,208,008 | 42.89% | 94.06% |
|  | Tr005-2 | 20,410,516 | 6,090,924,628 | 42.09% | 93.71% |
|  | Tr005-3 | 21,529,899 | 6,431,975,646 | 42.33% | 93.32% |
|  | PI251432-1 | 20,306,668 | 6,053,112,886 | 42.73% | 93.75% |
|  | PI251432-2 | 20,432,513 | 6,081,578,250 | 42.27% | 93.57% |
|  | PI251432-3 | 20,572,837 | 6,116,446,298 | 42.66% | 94.05% |
|  | Korla-1 | 20,843,087 | 6,230,881,378 | 42.04% | 93.55% |
|  | Korla-2 | 21,329,743 | 6,371,906,112 | 41.60% | 93.39% |
|  | Korla-3 | 21,460,936 | 6,413,813,828 | 41.83% | 93.35% |
| Salt stress | PI237292-1 | 21,921,639 | 6,516,799,048 | 41.96% | 94.28% |
|  | PI237292-2 | 21,418,900 | 6,390,303,514 | 41.67% | 94.13% |
|  | PI237292-3 | 20,535,172 | 6,120,739,188 | 42.25% | 94.21% |
|  | Tr005-1 | 21,708,203 | 6,462,836,866 | 42.20% | 94.13% |
|  | Tr005-2 | 21,694,756 | 6,444,037,454 | 42.19% | 94.47% |
|  | Tr005-3 | 23,897,573 | 7,103,695,036 | 42.25% | 93.92% |
|  | PI251432-1 | 22,197,880 | 6,630,000,368 | 41.92% | 93.75% |
|  | PI251432-2 | 19,708,617 | 5,883,657,588 | 42.30% | 93.66% |
|  | PI251432-3 | 23,532,098 | 6,994,039,076 | 42.02% | 94.61% |
|  | Korla-1 | 22,489,201 | 6,710,829,018 | 41.82% | 93.58% |
|  | Korla-2 | 22,214,707 | 6,610,129,964 | 41.84% | 94.06% |
|  | Korla-3 | 19,741,220 | 5,896,528,556 | 41.80% | 93.71% |


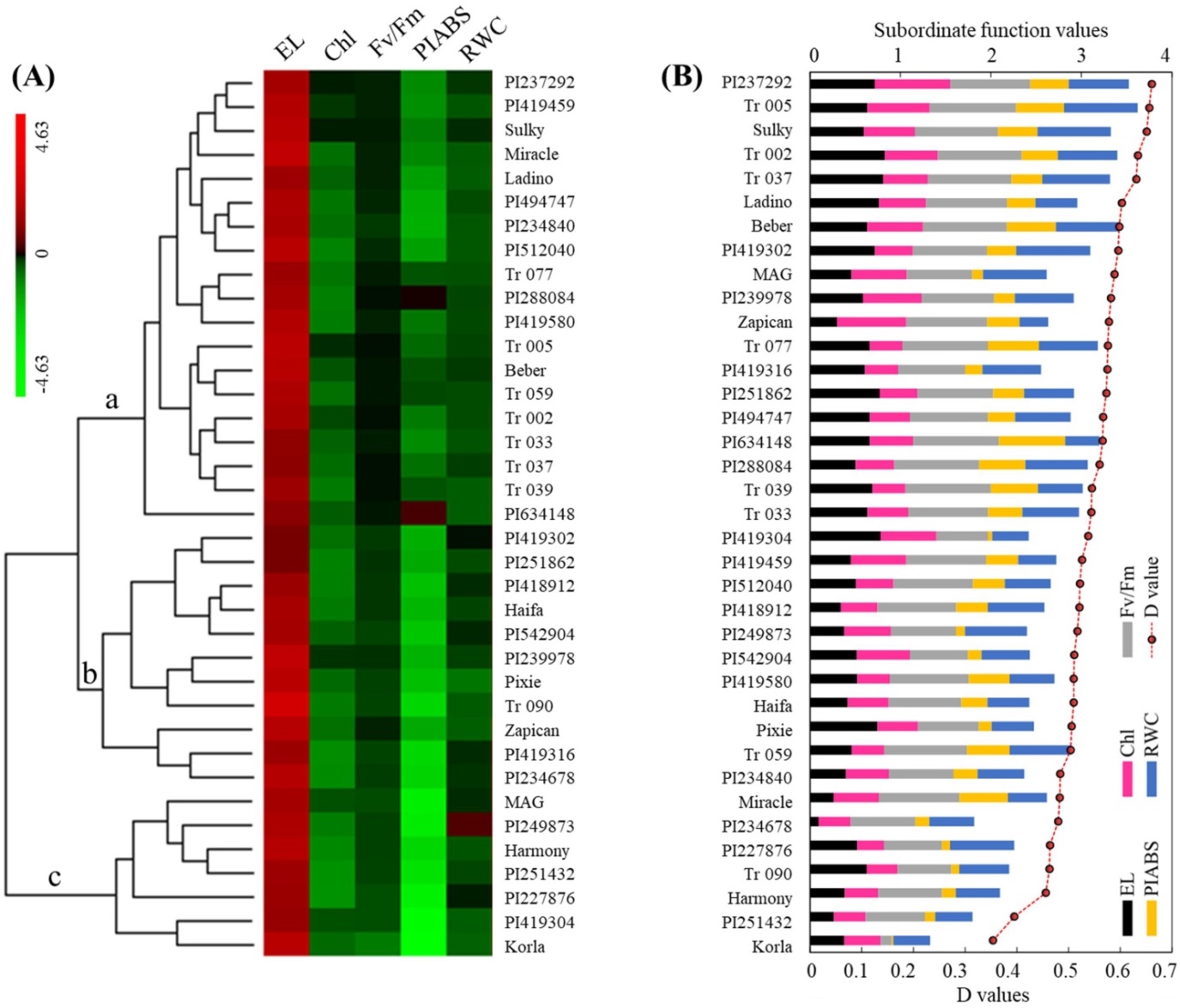


**Fig. S1** Changes in (A) heat map that was made by log2 FC (fold change of each parameter that was calculated by salt-stressed data in relation to the normal condition) and (B) the subordinate function values analysis of five different physiological parameters for comprehensive evaluation of salt tolerance among 37 white clover materials. EL, electrolyte leakage; Chl, chlorophyll; Fv/Fm, photochemical efficiency of PS II; PIABS, performance index on absorption basis; RWC, relative water content.


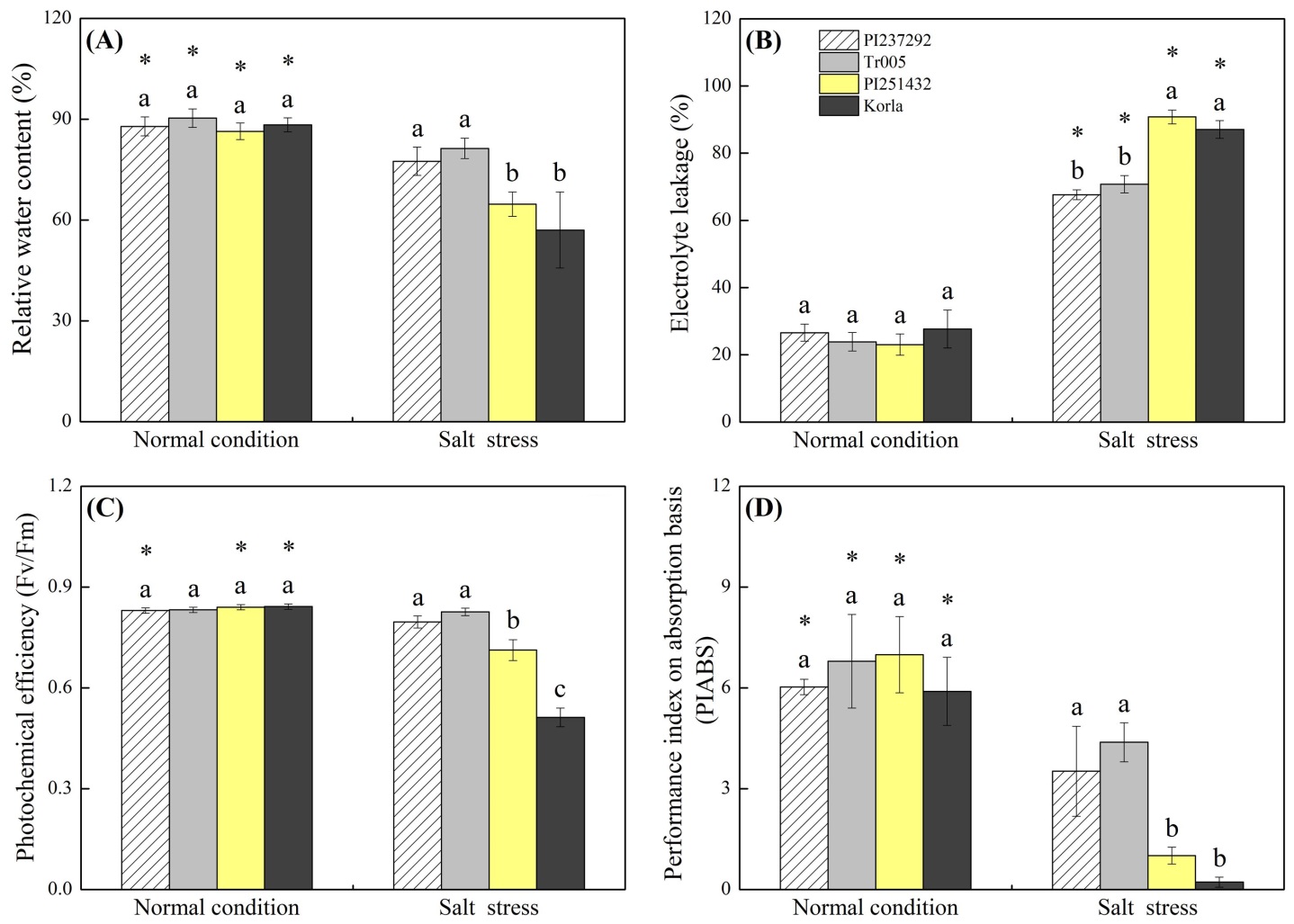


**Fig. S2** Changes in, (A) relative water content, (B) electrolyte leakage, (C) photochemical efficiency, and (D) performance index on absorption basis in leaves of four white clover materials (PI237292, Tr005, PI251432, and Korla) under normal and salt stress conditions. Vertical bars indicate ± standard error (SE) of mean (n = 4), and different letters above column indicate significant differences (*P* ≤ 0.05) under normal condition or salt stress. The “*” represents the significant difference for a specific genotype (PI237292, Tr005, PI251432, or Korla) between normal condition and salt stress.

#
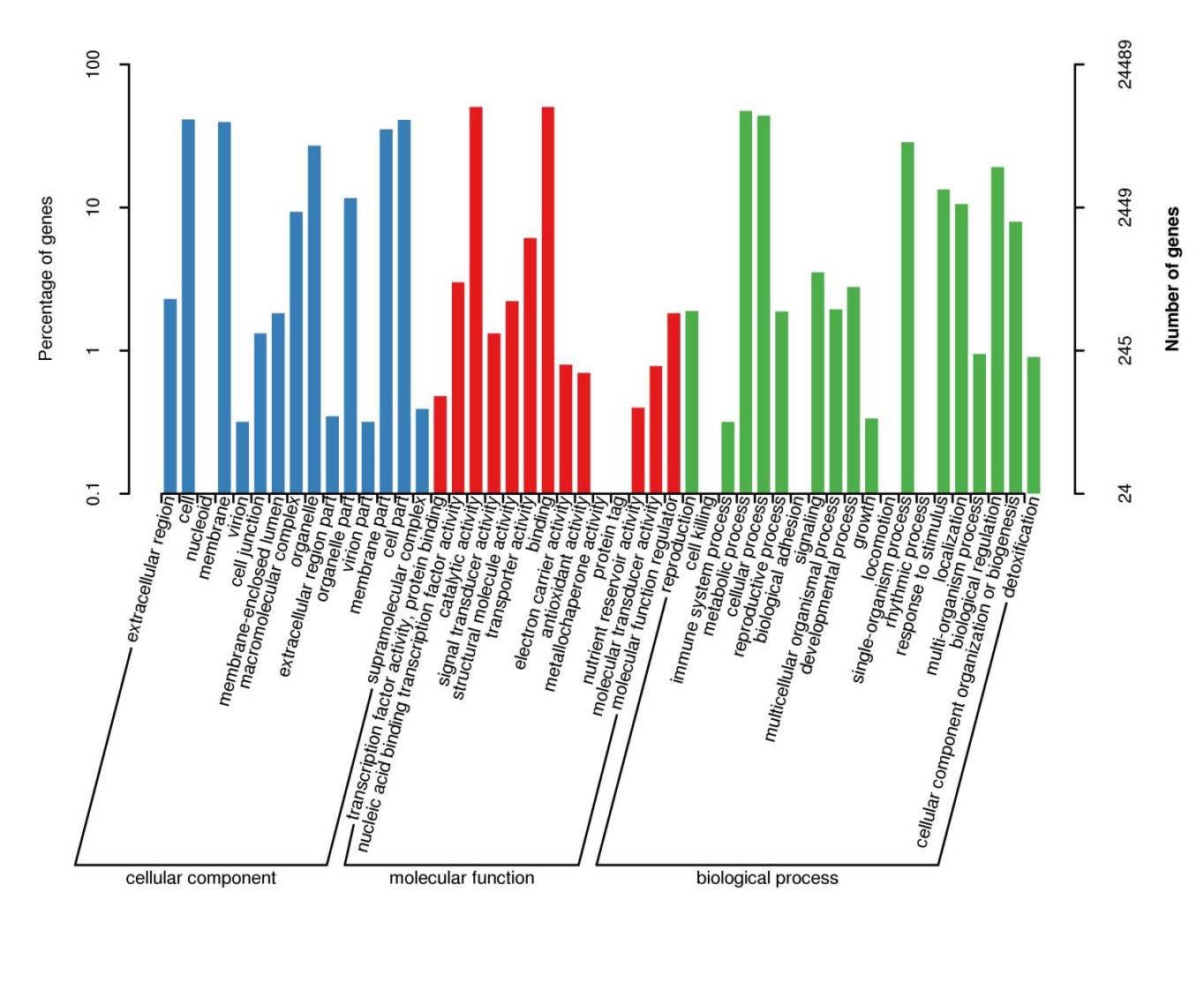


**Fig. S3** The Gene Ontology (GO) analysis of identified genes in leaves of white clover in response to salt stress.


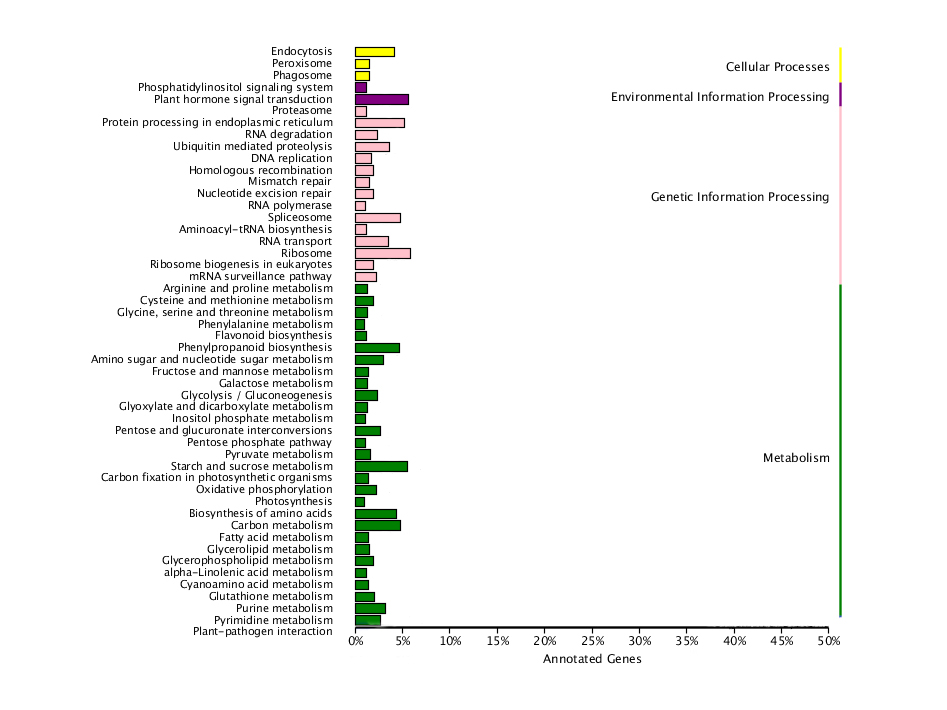


**Fig. S4** The Kyoto Encyclopedia of Genes and Genomes (KEGG) analysis of identified genes in leaves of white clover in response to salt stress.


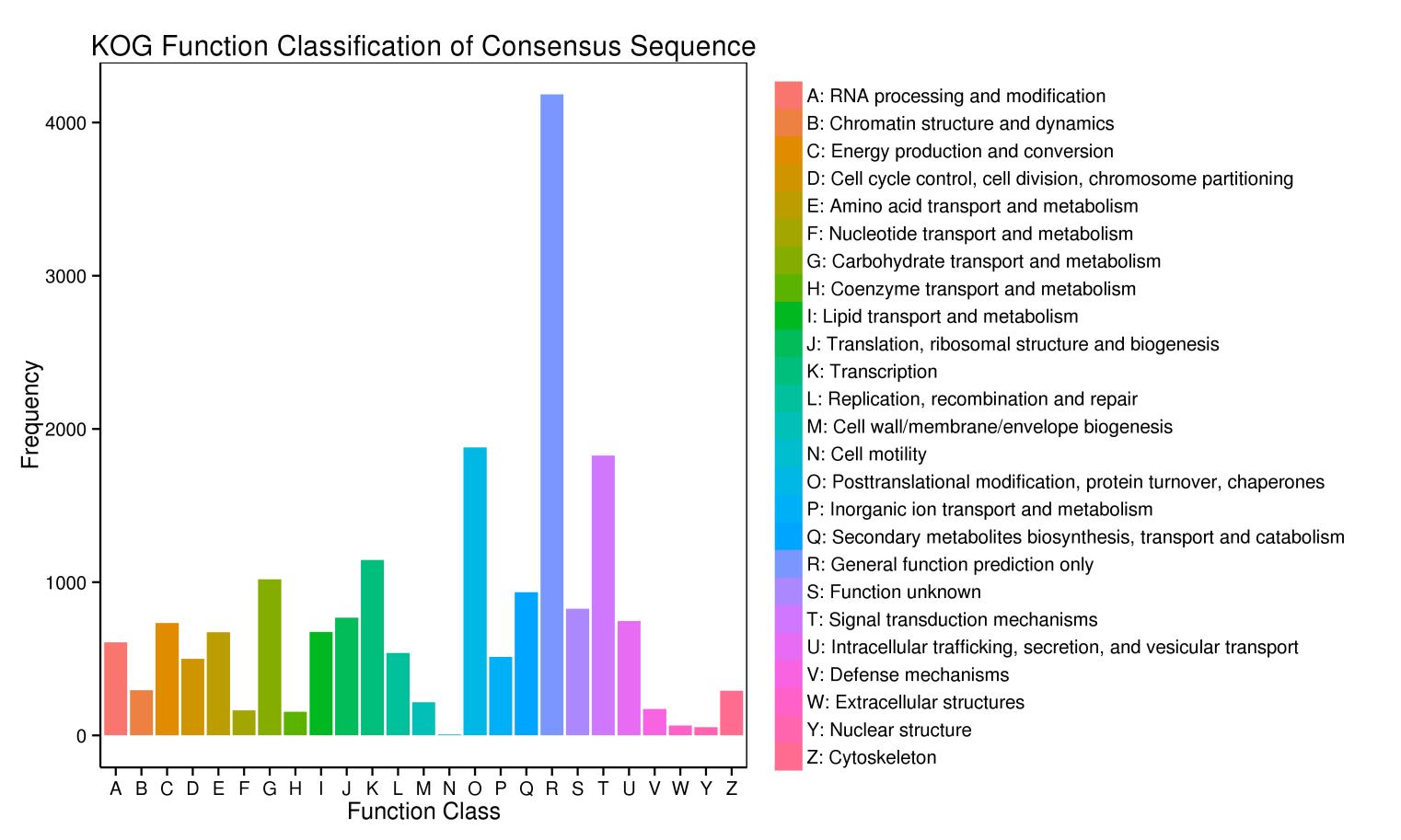


**Fig. S5** The Eukaryotic Orthologous Groups (KOG) function classification analysis of identified genes in leaves of white clover in response to salt stress.
